# Comparative expression profiles of microRNA in left and right atrial appendages from patients with rheumatic mitral valve disease exhibiting sinus rhythm or atrial fibrillation

**DOI:** 10.1186/1479-5876-12-90

**Published:** 2014-04-06

**Authors:** Hai Liu, Han Qin, Guang-xian Chen, Meng-ya Liang, Jian Rong, Jian-ping Yao, Zhong-kai Wu

**Affiliations:** 1 Second Department of Cardiac Surgery, First Affiliated Hospital of Sun Yat-Sen University, 58 Zhongshan II Road, Guangzhou 510080, China; 2 Department of Cardiopulmonary Bypass, First Affiliated Hospital of Sun Yat-Sen University, Guangzhou 510080, China; 3 Third Department of Cardiac Surgery, First Affiliated Hospital of Zhengzhou University, 1 Jianshedong Road, Zhengzhou 450052, China

**Keywords:** Atrial fibrillation, microRNA, Rheumatic mitral valve disease

## Abstract

**Background:**

The atrial fibrillation (AF) associated microRNAs (miRNAs) were found in the right atrium (RA) and left atrium (LA) from patients with rheumatic mitral valve disease (RMVD). However, most studies only focus on the RA; and the potential differences of AF-associated miRNAs between the RA and LA are still unknown. The aim of this study was to perform miRNA expression profiles analysis to compare the potential differences of AF-associated miRNAs in the right atrial appendages (RAA) and left atrial appendages (LAA) from RMVD patients.

**Methods:**

Samples tissues from the RAA and LAA were obtained from 18 RMVD patients (10 with AF) during mitral valve replacement surgery. From these tissues, miRNA expression profiles were created and analyzed using a human miRNA microarray. Then, the results were validated using qRT-PCR analysis for 12 selected miRNAs. Finally, potential targets of 10 validated miRNAs were predicted and their functions and potential pathways were analyzed using the miRFocus database.

**Results:**

In RAA, 65 AF-associated miRNAs were found and significantly dysregulated (i.e. 28 miRNAs were up-regulated and 37 were down-regulated). In LAA, 42 AF-associated miRNAs were found and significantly dysregulated (i.e. 22 miRNAs were up-regulated and 20 were down-regulated). Among these AF-associated miRNAs, 23 of them were found in both RAA and LAA, 45 of them were found only in RAA, and 19 of them were found only in LAA. Finally, 10 AF-associated miRNAs validated by qRT-PCR were similarly distributed in RAA and LAA; 3 were found in both RAA and LAA, 5 were found only in RAA, and 2 were found only in LAA. Potential miRNA targets and molecular pathways were identified.

**Conclusions:**

We have found the different distributions of AF-associated miRNAs in the RAA and LAA from RMVD patients. This may reflect different miRNA mechanisms in AF between the RA and LA. These findings may provide new insights into the underlying mechanisms of AF in RMVD patients.

## Background

Atrial fibrillation (AF) is the most common sustained arrhythmia in clinical practice and it is associated with pronounced morbidity, mortality, and socio-economic burden [1]. Effective therapy and prevention is crucial for the control of AF-related morbidity and mortality. However, to date, medical interventions for AF are relatively limited, because the precise mechanisms of AF have not been completely elucidated. Therefore, new methods leading to further insights into the underlying mechanisms of AF and potential novel mechanism-based therapeutic strategies are required [2].

MicroRNAs (miRNAs) are emerging as new regulators of gene expression at the post-transcriptional level and are shedding new light on the regulatory mechanisms underlying AF [3]. miRNAs are small, endogenous, single-stranded, non-coding RNAs of ∼22 nucleotides that bind target sequences in the 3′-untranslated region of target genes to induce mRNA instability or to inhibit translation [4]. Studies have shown that miRNAs regulate key genetic functions in cardiovascular biology and play an important role in the pathogenesis of cardiac diseases including cardiac development [5], cardiac hypertrophy/heart failure [6], myocardial infarction [7], myocardial ischemia-reperfusion injury [8], and arrhythmogenesis [9]. Recently, there are many studies indicating that miRNAs are involved in AF through their target genes [10-13].

AF often occurs concomitantly with other cardiovascular diseases such as hypertension, coronary artery disease, congestive heart failure, and valvular heart disease [14]. AF is also prevalent in rheumatic mitral valve disease (RMVD) [15]. RMVD is a major cardiovascular disease in developing countries where rheumatic fever is less well controlled, and is also a major clinical risk factor for AF [16]. Remodeling of the right atrium (RA) and left atrium (LA) associated with AF in RMVD patients are well established [17]. Studies have shown that miRNAs may be involved directly or indirectly in AF by modulating atrial remodelling [18]. Recently, the AF-associated miRNAs were respectively found in RA [19] and LA [20] from RMVD patients.

Morphological [21] and electrophysiological [17] differences have been demonstrated between the RA and LA, which at least in part, may reflect different mechanisms involved in AF between the RA and LA [22]. Thus, it is not surprising that AF-associated miRNAs of the RA may differ from those of the LA. However, most studies of AF-associated miRNAs focus on the RA; and the potential difference of AF-associated miRNAs between RA and LA are still unknown.

Thus, the aim of this study was to analyze miRNA expression profiles to compare the potential differences of AF-associated miRNAs in the RA and LA from RMVD patients who were either in an sinus rhythm (SR) or AF group.

## Methods

Approval was obtained from the human ethics committee of the first affiliated hospital of Sun Yat-sen University. The investigation complied with the principles that govern the use of human tissues outlined in the Declaration of Helsinki. All patients gave informed consent before participating in the study.

### Human tissue preparation

Tissue samples from the right atrial appendage (RAA) and left atrial appendage (LAA) were obtained from18 RMVD patients. 8 patients were in SR group and they did not have a history of AF; 10 patients were in AF group and they had documented arrhythmia for more than six months before surgery. The tissue samples were obtained at the time of the mitral valve replacement surgery, immediately snap frozen in liquid nitrogen, and stored at −80°C until used. The diagnosis of AF was made based on medical records and 12-lead electrocardiogram (ECG) findings. Patients with SR had no history of using antiarrhythmic drugs and were screened to ensure that they had never experienced AF [23]. Pre-operative color Doppler echocardiography was performed routinely on the patients. Preoperative functional status was recorded according to the New York Heart Association (NYHA) classifications.

### RNA isolation

Total RNA was extracted from human tissue samples (RAA and LAA) using TRIzol reagent (Invitrogen) according to the manufacturer’s protocol. The RNA quality of each sample was determined using an Agilent 2100 Bioanalyzer (Agilent Technologies; Santa Clara, CA, USA) and the sample was immediately stored at −80°C.

### MiRNA microarray processing and analysis

The miRNA microarray was processed by LC Sciences (Houston, TX, USA) as described previously [20]. In brief, the assay utilized 2 to 5 μg total RNA sample. The total RNA was size fractionated using a YM-100 Microcon centrifugal filter (Millipore, Billerica, MA) and RNA sequences with < 300 nt were isolated. These small RNA were then extended at 3′-end with a poly(A) tail using poly(A) polymerase, followed by ligation of an oligonucleotide tag to the poly(A) tail for later fluorescent dye staining. Hybridization was performed overnight on a μParaflo™ microfluidic chip using a micro-circulation pump (Atactic Technologies, Inc, Houston, TX). Each microfluidic chip contained detection probes and control probes. The detection probes were made in situ by photogenerated reagent (PGR) chemistry. These probes consisted of a chemically modified nucleotide coding sequence complementary to the target microRNA (all 1,921 human miRNAs listed in the Sanger’s miRNAmiRBase, Release 18.0, http://microrna.sanger.ac.uk/sequences/) and a spacer segment of polyethylene glycol to extend the coding sequences away from the substrate. The hybridization melting temperatures were balanced by chemical modifications of the detection probes. Hybridization was performed using 100 μL of 6× SSPE buffer (0.90 M NaCl, 60 mM Na2HPO4, 6 mM EDTA, pH 6.8) containing 25% formamide at 34°C. Fluorescence labeling with tag specific Cy5 dye was used for after-hybridization detection. An Axon GenePix 4000B Microarray Scanner (Molecular Device, Union City, CA) was used to collect the fluorescent images, which were then digitized using Array-Pro Image Analysis software (Media Cybernetics, Bethesda, MD). Each miRNA was analyzed two times and the controls were repeated 4–16 times.

The miRNA microarray analysis was also performed at LC Sciences. The microarray data were analyzed by subtracting the background and then the signals were normalized using a locally weighed regression (LOWESS) filter as reported previously [24]. Detectable miRNAs were selected based on the following criteria: signal intensity higher than 3× the background standard deviation and spot CV < 0.5 (where CV = standard deviation/signal intensity). When repeating probes were present on an array, a transcript was listed as detectable only if the signals from at least 50% of the repeating probes were above detection level. To identify miRNAs whose expression differs among the groups, statistic analysis was performed and the *P*-values of the *t*-test were also calculated. The ratio of two samples was calculated and expressed in log_2_^scale (balanced)^ for each miRNA. The miRNAs were then sorted according to their differential ratios.

### Quantitative real-time PCR (qRT-PCR) of miRNA expression

To validate the microarray results in the study, a stem-loop qRT-PCR based on SYBR Green I was performed on differentially expressed miRNAs. The primers used are listed in Additional file 1. Total RNA was isolated using the TRIzol Reagent (Invitrogen) as previously described. A single-stranded cDNA for a specific miRNA was generated by reverse transcription of 250 ng of total RNA using a miRNA-specific stem-looped RT primer. Briefly, a reverse transcription reaction mixture contained 250 ng of total RNA, 0.5 μl of 2 μM stem-loop RT primer, 1.0 μl of 5× RT buffer, 0.25 μl of 10 mM each dNTPs, 0.25 μl of 40 U/μlRNase inhibitor and 0.5 μl of 200 U/μl M-MLV. An Eppendorf Mastercycler® (Eppendorf, Hamburg, Germany) was used to conduct the reverse transcription reaction at the following temperature conditions: 42°C for 60 min, 70°C for 15 min and finally held at 4°C.

Following the reverse transcription reaction, qRT-PCR was performed using an ABI PRISM® 7900HT sequence-detection system (Applied Biosystems, Foster City, CA, USA) with the Platinum SYBR Green qPCR SuperMix-UDG (Invitrogen). According to the manufacturer’s instructions, the PCR reaction (a total of 20 μl) contained 0.5 μl of RT product, 10 μl of 2× SYBR Green Mix, 0.4 μl of ROX, 0.8 μl of 10 μM primer mix, and 8.3 μl of nuclease-free water. The reaction protocol was as follows: 95°C for 2 min, followed by 40 amplification cycles of 95°C for 15 s, and 60°C for 30 s.

In qRT-PCR analysis, the relative expression level for each miRNA was calculated using a comparative cycle threshold 2^-ΔΔCt^ method for three independent experiments [25]. RNU6B was used as an internal control for normalizing the results.

### Target prediction and function analysis

We used the human miRNA information database miRFocus (http://mirfocus.org/ Version 2.1) to predict potential human miRNA target genes. The miRFocus provides a full gene description and functional analysis for each target gene by combining the predicted target genes from other databases (TargetScan, miRanda, PicTar, MirTarget and microT). The greater the number of databases predicting that a gene would be a target, the more likely the miRNA-mRNA interaction would be relevant [26]. In this study, we have included genes that were predicted by two or more databases. The miRFocus program also identifies miRNA-enriched pathways, incorporating those from the Kyoto Encyclopedia of Genes and Genomes (KEGG), Biocarta, and Gene Ontology (GO) databases, using Fisher’s exact test.

### Statistical analyses

All data are presented as mean ± standard deviation and analyzed by paired *t*-test. For microarray results, miRNAs with *P*-values < 0.1 and |log_2_^ratio^| > 0.5 were considered to be significantly differentially expressed, while *P* < 0.05 was considered statistically significant for qRT-PCR analysis.

## Results

### Clinical characteristics of the SR and AF patients

Tissues from both RAA and LAA were obtained from each patient.There were no significant differences in terms of age, gender or NYHA classifications between the SR and AF groups. Pre-operative color Doppler echocardiography showed that the LA size of the patients with AF was significantly greater than patients with SR as previously reported [27]. There were no differences in the left ventricular ejection fraction (LVEF) between the groups (Table 1).

**Table 1:** Clinical characteristics of the SR and AF patients

	**SR (n = 8)**	**AF (n = 10)**
Gender (male/female)	5/3	5/5
Age (years)	50.16 ± 6.88	51.42 ± 7.12
LA size (mm)	43.31 ± 3.23	57.65 ± 5.08*
LVEF (%)	61.34 ± 5.66	58.71 ± 3.81
NYHA class	II (6/8) / III (2/8)	II (7/10) / III (3/10)

*P < 0.05 (comparing to the SR patients).

### miRNA expression profiles in SR-RAA, SR-LAA, AF-RAA, AF-LAA

**Figure 1:**
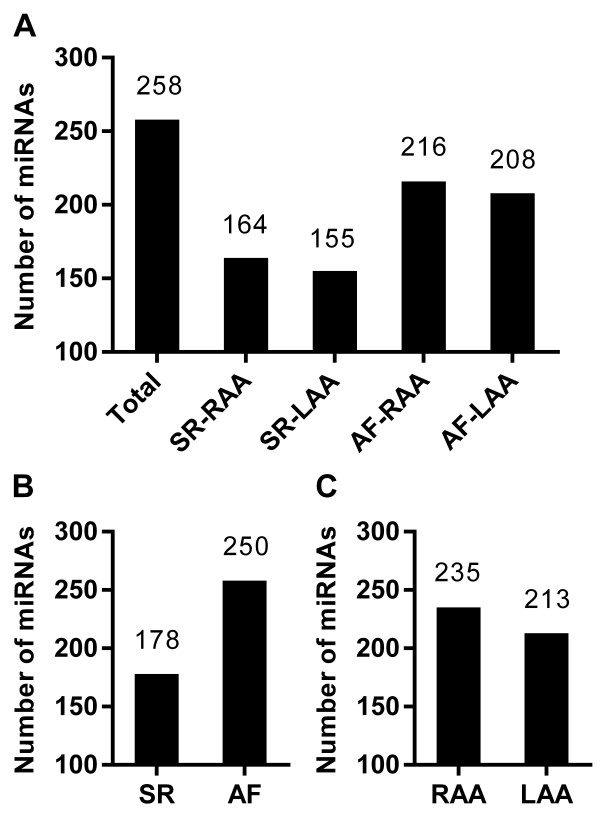
**The numbers of miRNAs detected in SR-RAA, SR-LAA, AF-RAA and AF-LAA using miRNA microarray assay. (A)** A total of 258 miRNAs were detected (in SR-RAA, SR-LAA, AF-RAA, or AF-LAA). In the SR-RAA, SR-LAA, AF-RAA, and AF-LAA groups 164, 155, 216, and 208 miRNAs were expressed, respectively. **(B)** 178 miRNAs were expressed in SR patients (SR-RAA or SR-LAA), while 250 miRNAs were expressed in AF patients (AF-RAA or AF-LAA). **(C)** 235 miRNAs were expressed in RAA tissues (SR-RAA or AF-RAA), while 213 miRNAs were expressed in LAA tissues (SR-LAA or AF-LAA).

Of the 1,898 human miRNAs analyzed, a total of 258 miRNAs were detected (in SR-RAA, SR-LAA, AF-RAA, or AF-LAA). In the SR-RAA, SR-LAA, AF-RAA, and AF-LAA groups 164, 155, 216, and 208 miRNAs were expressed, respectively (Figure 1A). Among these, 132 miRNAs were detected in all groups. A number of miRNAs were expressed only in one of the four groups—6 in the SR-RAA group, 2 in the SR-LAA group, 40 in the AF-RAA group, and 19 in the AF-LAA group.

Among the 258 miRNAs, 178 miRNAs were expressed in SR patients (SR-RAA or SR-LAA), while 250 miRNAs were expressed in AF patients (AF-RAA or AF-LAA). AF resulted in greater expression of miRNAs than SR (Figure 1B). A total of 235 miRNAs were expressed in RAA tissues (SR-RAA or AF-RAA), while 213 miRNAs were expressed in LAA tissues (SR-LAA or AF-LAA). RAA tissues had a larger number of miRNAs expressed (Figure 1C).

**Figure 2:**
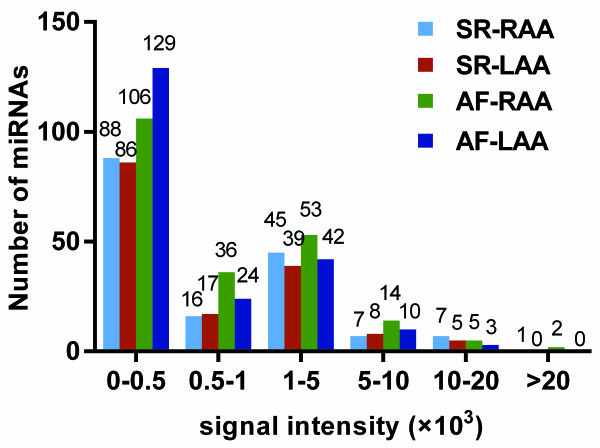
**Signal distributions of all detected miRNAs in SR-RAA, SR-LAA, AF-RAA and AF-LAA.** The signal intensities of most of these miRNAs were low (0–500 units), with only 23 miRNAs showing intensities greater than 10000 units.

For most of the detected miRNAs, the expression levels were low, which was evident by their low signal intensities (less than 500 units) during microarray analysis (Figure 2). Of the 164 miRNAs detected in SR-RAA tissues, 88 miRNAs emitted signals less than 500 units, while only 8 miRNAs produced signals above 10,000 units. Of the 155 miRNAs detected in SR-LAA tissues, 86 miRNAs emitted signals less than 500 units, while only 5 miRNAs produced signals above 10,000 units. Of the 216 miRNAs detected in AF-RAA tissues, 106 miRNAs emitted signals less than 500 units, while only 7 miRNAs produced signals above 10,000 units. Of the 208 miRNAs detected in AF-LAA tissues, 129 produced signals below 500 units, while only 3 produced signals above 10,000 units.

The signal intensities of the miRNAs expressed in only one of the four groups were not high enough to consider them differentially expressed among groups, and hence were not be considered for further analysis.

### miRNA expression profiles changes associated with AF in RAA tissue

**Figure 3:**
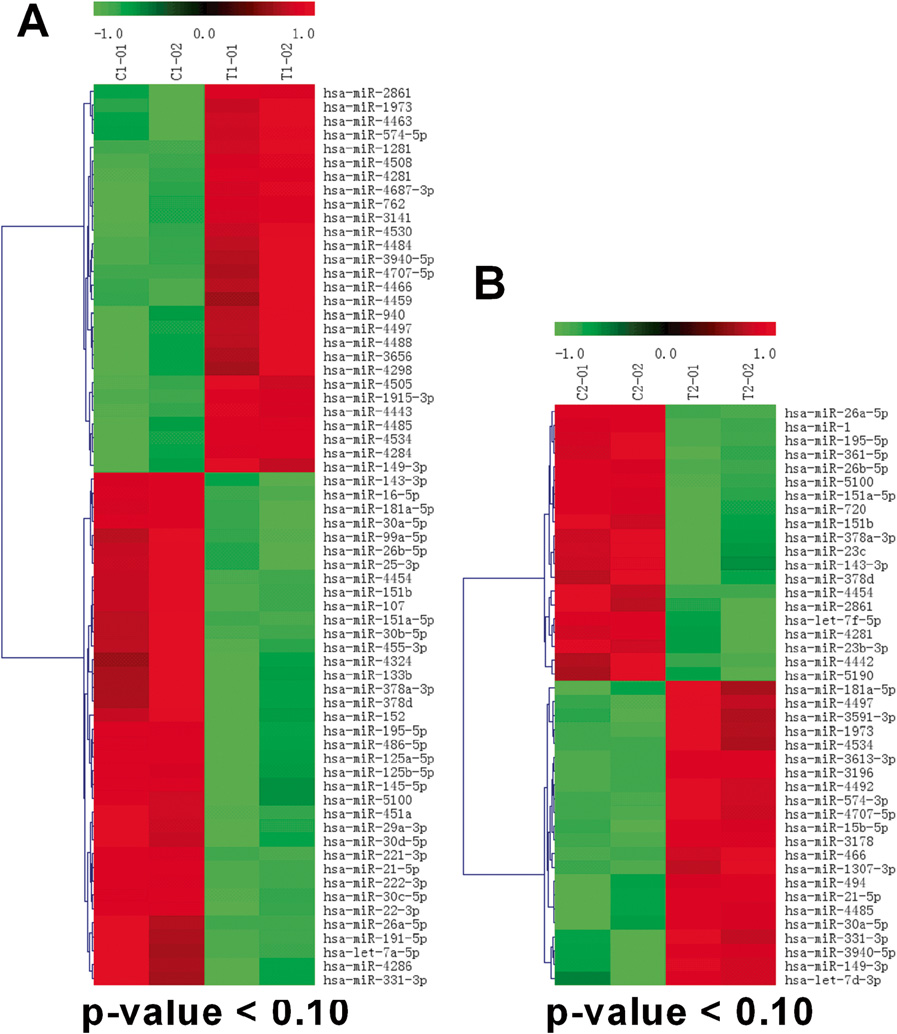
**Heat map showing the miRNAs significantly dysregulated in RAA and LAA tissues. (A)** Heat map showing the expression of dysregulated miRNAs in RAA tissues (AF-RAA vs SR-RAA). **(B)** Heat map showing the expression of dysregulated miRNAs in LAA tissues (AF-LAA vs SR-LAA).

The SR-RAA group expressed 164 miRNAs, while the AF-RAA group expressed 216 miRNAs. Along with the number of detectable miRNAs, the expression levels of these miRNAs were also significantly different. Statistical analysis showed that 65 of these miRNAs were significantly dysregulated between AF-RAA and SR-RAA. Among these AF-associated miRNAs, 28 were up-regulated and 37 were down-regulated (Table 2 and Figure 3A).

**Table 2:** miRNA expression differences between AF-RAA and SR-RAA

**miRNA**	**SR-RAA signal**	**AF-RAA signal**	**Log**_**2**_^**(AF-RAA/SR-RAA)**^	***P*-value**
Up-regulated (n = 28*)				
hsa-miR-4687-3p	378	1654	2.13	3.86E-02
hsa-miR-4485	119	518	2.12	6.98E-02
hsa-miR-4484	879	3609	2.04	5.09E-02
hsa-miR-762	142	544	1.94	4.24E-02
hsa-miR-3940-5p	489	1740	1.83	6.45E-02
hsa-miR-149-3p	107	373	1.81	7.71E-02
hsa-miR-4707-5p	371	1295	1.80	6.57E-02
hsa-miR-4281	480	1524	1.67	2.85E-02
hsa-miR-574-5p	279	883	1.66	6.56E-02
hsa-miR-1281	229	713	1.64	2.26E-02
hsa-miR-3141	883	2687	1.60	4.32E-02
hsa-miR-4488	1049	2915	1.47	7.12E-02
hsa-miR-1973	876	2203	1.33	5.07E-02
hsa-miR-4463	241	587	1.28	5.99E-02
hsa-miR-4505	213	504	1.24	3.66E-02
hsa-miR-4466	1286	3026	1.23	5.73E-02
hsa-miR-940	280	629	1.17	8.74E-02
hsa-miR-4459	2647	5576	1.08	8.57E-02
hsa-miR-2861	1081	2265	1.07	4.66E-02
hsa-miR-4534	105	208	0.99	4.21E-02
hsa-miR-3656	2615	5009	0.94	8.13E-02
hsa-miR-4530	2265	4225	0.90	4.38E-02
hsa-miR-4443	182	326	0.84	2.88E-02
hsa-miR-4284	159	276	0.80	5.12E-02
hsa-miR-4508	1836	2991	0.70	2.36E-02
hsa-miR-1915-3p	1805	2917	0.69	2.12E-02
hsa-miR-4298	1478	2314	0.65	8.97E-02
hsa-miR-4497	3651	5392	0.56	6.18E-02
Down-regulated (n = 37*)				
hsa-miR-451a	2775	97	−4.83	3.47E-02
hsa-miR-29a-3p	778	156	−2.32	5.04E-02
hsa-miR-99a-5p	645	162	−1.99	5.83E-02
hsa-miR-25-3p	386	101	−1.93	4.99E-02
hsa-miR-486-5p	632	167	−1.92	5.85E-02
hsa-miR-16-5p	683	186	−1.88	2.05E-02
hsa-miR-455-3p	220	61	−1.85	5.70E-02
hsa-miR-222-3p	389	116	−1.75	1.34E-02
hsa-miR-195-5p	902	290	−1.64	5.19E-02
hsa-miR-221-3p	252	82	−1.62	1.13E-02
hsa-miR-22-3p	302	104	−1.54	3.02E-02
hsa-miR-331-3p	216	78	−1.47	9.03E-02
hsa-miR-4324	1409	540	−1.38	9.86E-02
hsa-miR-30c-5p	2822	1107	−1.35	2.87E-02
hsa-miR-378d	171	69	−1.32	8.68E-02
hsa-miR-125a-5p	3506	1488	−1.24	6.63E-02
hsa-miR-151a-5p	622	267	−1.22	5.18E-02
hsa-miR-143-3p	1732	746	−1.22	4.85E-02
hsa-miR-151b	556	253	−1.13	3.69E-02
hsa-miR-4454	1276	582	−1.13	3.42E-02
hsa-miR-145-5p	7284	3352	−1.12	8.73E-02
hsa-miR-378a-3p	477	221	−1.11	8.70E-02
hsa-miR-30b-5p	1857	869	−1.10	5.33E-02
hsa-miR-26b-5p	1342	638	−1.07	5.00E-02
hsa-miR-133b	3912	1868	−1.07	9.81E-02
hsa-miR-107	592	284	−1.06	3.95E-02
hsa-miR-152	293	141	−1.05	7.94E-02
hsa-miR-30a-5p	409	201	−1.03	2.72E-02
hsa-miR-125b-5p	7602	3820	−0.99	7.43E-02
hsa-miR-4286	243	128	−0.92	8.45E-02
hsa-miR-191-5p	1441	767	−0.91	7.19E-02
hsa-miR-26a-5p	9621	5219	−0.88	6.04E-02
hsa-miR-21-5p	633	351	−0.85	8.92E-03
hsa-miR-30d-5p	1225	692	−0.82	5.79E-02
hsa-miR-5100	458	274	−0.74	8.76E-02
hsa-miR-181a-5p	998	613	−0.70	3.23E-02
hsa-let-7a-5p	16804	10484	−0.68	7.85E-02

*Expression levels of miRNAs are described as up-regulated or down-regulated in AF-RAA relative to those in SR-RAA.

### miRNA expression profiles changes associated with AF in LAA tissue

The SR-LAA group expressed 155 miRNAs, while the AF-LAA group expressed 208 miRNAs. Along with the number of detectable miRNAs, significant differences also existed in the expression levels of these miRNAs. Statistical analysis showed that 42 miRNAs were significantly dysregulated in AF-LAA relative to SR-LAA. Among these AF-associated miRNAs, 22 were up-regulated and 20 were down-regulated (Table 3 and Figure 3B)

**Table 3:** miRNA expression differences between AF-LAA and SR-LAA

**miRNA**	**SR-LAA signal**	**AF-LAA signal**	**Log**_**2**_^**(AF-LAA/SR-LAA)**^	***P*-value**
Up-regulated (n = 22*)				
hsa-miR-3613-3p	294	3063	3.38	1.07E-02
hsa-miR-494	296	1881	2.67	5.89E-02
hsa-miR-3591-3p	539	2069	1.94	7.14E-02
hsa-miR-4485	80	284	1.83	5.03E-02
hsa-miR-574-3p	4478	13516	1.59	2.41E-02
hsa-miR-466	1183	3343	1.50	2.31E-02
hsa-miR-4492	334	834	1.32	2.73E-02
hsa-let-7d-3p	146	344	1.23	9.94E-02
hsa-miR-4707-5p	387	857	1.15	2.92E-02
hsa-miR-4534	79	172	1.12	6.66E-02
hsa-miR-3940-5p	397	803	1.02	5.71E-02
hsa-miR-3178	185	374	1.01	1.97E-02
hsa-miR-15b-5p	383	723	0.92	3.04E-02
hsa-miR-21-5p	442	819	0.89	4.66E-02
hsa-miR-3196	1383	2477	0.84	1.34E-02
hsa-miR-1307-3p	82	145	0.83	5.28E-02
hsa-miR-331-3p	140	247	0.82	6.46E-02
hsa-miR-149-3p	96	163	0.77	6.79E-02
hsa-miR-181a-5p	1057	1613	0.61	8.50E-02
hsa-miR-30a-5p	249	376	0.59	6.94E-02
hsa-miR-1973	745	1123	0.59	6.02E-02
hsa-miR-4497	4574	6546	0.52	4.68E-02
Down-regulated (n = 20*)				
hsa-miR-1	12740	3079	−2.05	1.94E-02
hsa-miR-26b-5p	794	198	−2.00	2.30E-02
hsa-miR-4454	1262	488	−1.37	3.93E-02
hsa-miR-361-5p	652	259	−1.33	3.53E-02
hsa-miR-151a-5p	694	302	−1.20	3.90E-02
hsa-miR-26a-5p	9702	4314	−1.17	7.22E-03
hsa-miR-378a-3p	683	307	−1.15	7.26E-02
hsa-miR-5190	311	141	−1.14	9.06E-02
hsa-miR-5100	775	363	−1.10	2.89E-02
hsa-miR-151b	609	288	−1.08	6.25E-02
hsa-miR-4442	350	169	−1.05	6.69E-02
hsa-miR-2861	2103	1141	−0.88	6.50E-02
hsa-miR-143-3p	1380	778	−0.83	9.49E-02
hsa-miR-378d	197	112	−0.82	7.07E-02
hsa-miR-23c	939	583	−0.69	8.28E-02
hsa-miR-195-5p	612	385	−0.67	2.21E-02
hsa-miR-720	952	619	−0.62	4.67E-02
hsa-miR-4281	836	545	−0.62	6.92E-02
hsa-let-7f-5p	10225	7040	−0.54	5.58E-02
hsa-miR-23b-3p	12468	8685	−0.52	7.24E-02

*Expression levels of miRNAs are described as up-regulated or down-regulated in AF-LAA relative to those in SR-LAA.

### Comparison of AF-associated miRNAs between RAA and LAA tissues

A total of 84 AF-associated miRNAs were found (either in RAA or LAA tissues): 65 AF-associated miRNAs were found in RAA tissues, while 42 AF-associated miRNAs were found in LAA tissues. Among these, 23 AF-associated miRNAs were found both in RAA and LAA, while 45 AF-associated miRNAs were found only in RAA, and 19 AF-associated miRNAs were found only in LAA (Table 4).

**Table 4:** Comparison of AF-associated miRNAs between RAA and LAA

	**Number**	**AF-associated miRNAs**
Both in RAA and LAA	23	hsa-miR-143-3p, hsa-miR-149-3p, hsa-miR-151a-5p, hsa-miR-151b, hsa-miR-181a-5p, hsa-miR-195-5p, hsa-miR-1973, hsa-miR-21-5p, hsa-miR-26a-5p, hsa-miR-26b-5p, hsa-miR-2861, hsa-miR-30a-5p, hsa-miR-331-3p, hsa-miR-378a-3p, hsa-miR-378d, hsa-miR-3940-5p, hsa-miR-428, hsa-miR-4454, hsa-miR-4485, hsa-miR-4497, hsa-miR-4534, hsa-miR-4707-5p, hsa-miR-5100
Only in RAA	42	hsa-let-7a-5p, hsa-miR-107, hsa-miR-125a-5p, hsa-miR-125b-5p, hsa-miR-1281, hsa-miR-133b, hsa-miR-145-5p, hsa-miR-152, hsa-miR-16-5p, hsa-miR-1915-3p, hsa-miR-191-5p, hsa-miR-221-3p, hsa-miR-222-3p, hsa-miR-22-3p, hsa-miR-25-3p, hsa-miR-29a-3p, hsa-miR-30b-5p, hsa-miR-30c-5p, hsa-miR-30d-5p, hsa-miR-3141, hsa-miR-3656, hsa-miR-4284, hsa-miR-4286, hsa-miR-4298, hsa-miR-4324, hsa-miR-4443, hsa-miR-4459, hsa-miR-4463, hsa-miR-4466, hsa-miR-4484, hsa-miR-4488, hsa-miR-4505, hsa-miR-4508, hsa-miR-451a, hsa-miR-4530, hsa-miR-455-3p, hsa-miR-4687-3p, hsa-miR-486-5p, hsa-miR-574-5p, hsa-miR-762, hsa-miR-940, hsa-miR-99a-5p
Only in LAA	19	hsa-let-7d-3p, hsa-let-7f-5p, hsa-miR-1, hsa-miR-1307-3p, hsa-miR-15b-5p, hsa-miR-23b-3p, hsa-miR-23c, hsa-miR-3178, hsa-miR-3196, hsa-miR-3591-3p, hsa-miR-3613-3p, hsa-miR-361-5p, hsa-miR-4442, hsa-miR-4492, hsa-miR-466, hsa-miR-494, hsa-miR-5190, hsa-miR-574-3p, hsa-miR-720

### Validation of the miRNA microarray data by qRT-PCR

**Figure 4:**
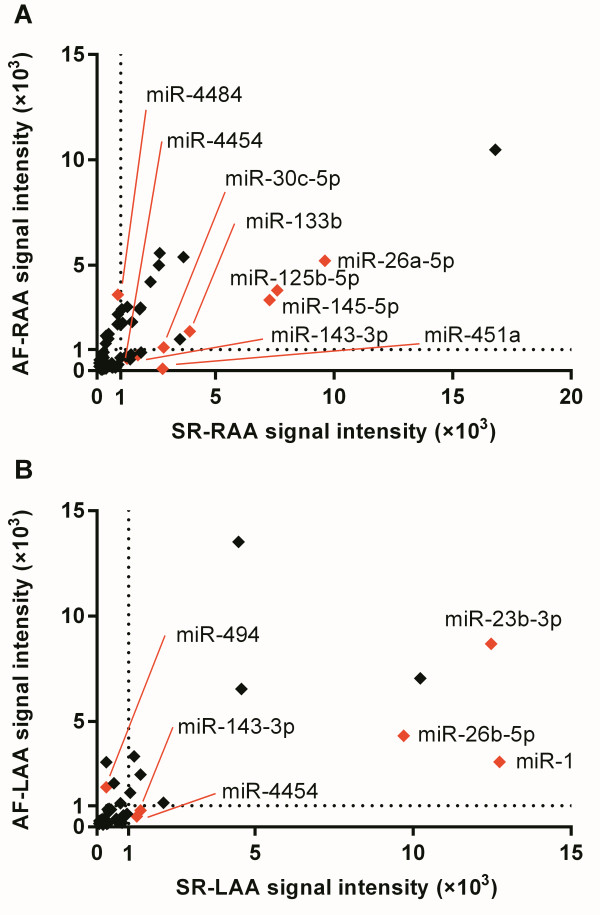
**Comparison of signal intensities of miRNAs significantly dysregulated in RAA and LAA tissues.** The signal intensities of miRNAs expressed in the SR group (x-axis) and AF group (y-axis) were compared. The miRNAs with the higher signal intensities (>1000 units) either on the x-axis or y-axis were labelled red. **(A)** AF-RAA vs SR-RAA. **(B)** AF-LAA vs SR-LAA.

To validate the data obtained from the miRNA microarray, qRT-PCR was performed on 12 AF-associated miRNAs. The AF-associated miRNAs were selected for further analysis using qRT-PCR based on the following criteria: at least one group (SR or AF group) had a signal intensity >1000 units in the RAA or LAA tissues. Finally, we selected 9 AF-associated miRNAs from RAA (Figure 4A) and 6 AF-associated miRNAs from LAA (Figure 4B). Among these: 3 miRNAs (i.e. miR-26a-5p, miR-143-3p, and miR-4454) were expressed in both RAA and LAA; 6 miRNAs (i.e. miR-30c-5p, miR-125b-5p, miR-133b, miR- 145-5p, miR-451a, and miR-4484) were expressed in only RAA; and 3 miRNAs (i.e. miR-1, miR-23b-3p, and miR-494) were expressed in only LAA.

**Figure 5:**
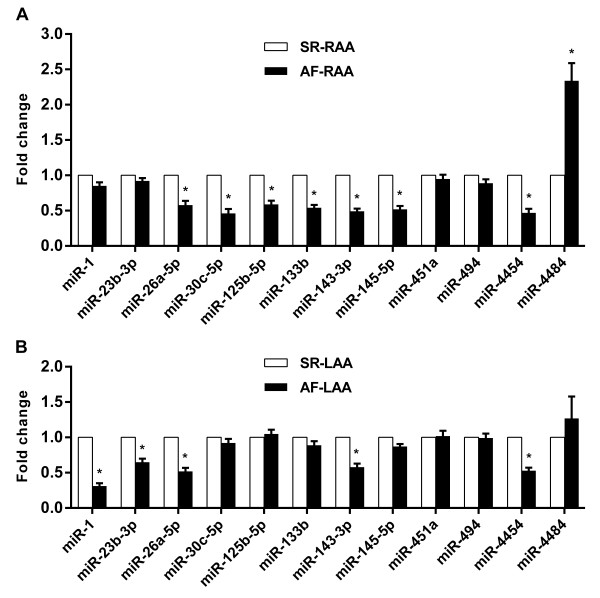
**Validation of the miRNA microarray data using qRT-PCR.** RNU6B was used as an internal control for normalizing the results. Data are reported as mean ± standard deviation for three independent experiments. Statistically significant differences between the two groups are indicated by *P < 0.05, compared with SR-RAA **(A)** or SR-LAA **(B)**.

According to the qRT-PCR data, miR-26a-5p, miR-143-3p, miR-4454 were AF-associated miRNAs found in both RAA and LAA tissues, while miR-30c-5p, miR-125b-5p, miR-133b, miR-145-5p, miR-4484 were AF-associated miRNAs found in only RAA tissues and miR-1, miR-23b-3p were found only in LAA tissues (Figure 5). Our qRT-PCR data validated the results for all of these miRNAs except miR-451a and miR-494. miR-451a was an AF-associated miRNA found only in RAA in the array data (Table 4), but was neither found in the RAA nor LAA when assayed using qRT-PCR (Figure 5). miR-494 was AF-associated miRNA found only in the LAA from the array data (Table 4), but was neither found in the RAA nor LAA when assayed using qRT-PCR (Figure 5).

### Comparing expression of AF-associated miRNAs in RAA and LAA tissues from RMVD patients with SR

**Figure 6:**
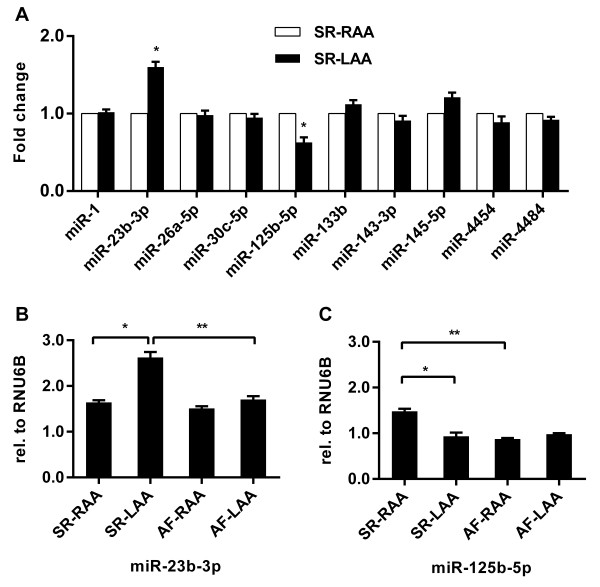
**Comparison of validated AF-associated miRNAs expression level between RAA and LAA in SR status.** Data are reported as mean ± standard deviation for three independent experiments, *P < 0.05, compared with SR-RAA **(A,B,C)**; **P < 0.05, compared with SR-LAA **(B)** or SR-RAA **(C)**.

We identified 10 AF-associated miRNAs using the microarray and qRT-PCR techniques. These AF-associated miRNAs had different tissue distributions between the RAA and LAA (Table 4, Figure 5). We wanted to know whether expressions of these miRNAs were different between the RAA and LAA tissues based on SR status. So we next compared expression levels of these miRNAs between the SR-RAA and SR-LAA groups. The expression levels of most of the AF-associated miRNAs were identical between RAA and LAA in SR patients, with the exception of miR-23b-3p and miR-125b-5p (Figure 6A). miR-23b-3p was more highly expressed in the SR-LAA group than in SR-RAA, and miR-125b-5p was more highly expressed in the SR-RAA group. Moreover, we compared the expression level of miR-23b-3p and miR-125b-5p among four groups (i.e. SR-RAA, SR-LAA, AF-RAA, and AF-LAA). In LAA tissues, the expression level of miR-23b-3p was down-regulated in the AF group relative to the SR group. In RAA tissues, the expression level of miR-23b-3p did not change between the SR group and AF group (Figure 6B). Meanwhile, a change in miR-125b-5p expression between SR and AF groups occurred only in RAA tissues, where miR-125b-5p was more highly expressed in SR group (Figure 6C).

### Prediction of putative target genes and pathways of the AF-associated miRNAs

To determine the probable biological function of the AF-associated miRNAs, we predicted the putative targets and pathways of 10 validated miRNAs (i.e. miR-1, miR-23b-3p, miR-26a-5p, miR-30c-5p, miR-125b-5p, miR-133b, miR-143-3p, miR-145-5p, miR-4454, and miR-4484) using the miRFocus database.

Most of these miRNAs were predicted by 5 target prediction databases except miR-4454 and miR-4484. miR-4454 cannot be predicted using 5 target prediction databases, while, miR-4484 can be predicted using 2 target prediction databases. Numerous putative target genes and pathways were identified for these miRNAs except miR-4454. Many of these predicted targets have been experimentally validated (Table 5, Additional file 2).

**Table 5:** Prediction of putative target genes and pathways of the validated miRNAs

**miRNAs**	**target gene**		**GO term**	**KEGG pathways**	**Target prediction database**
	**Total**	**Experimental validated**			
miR-1	135	68	101	9	miRanda, MirTarget, microT, PicTar, TargetScan
miR-23b-3p	296	8	91	5	miRanda, MirTarget, microT, PicTar, TargetScan
miR-26a-5p	210	7	62	11	miRanda, MirTarget, microT, PicTar, TargetScan
miR-30c-5p	365	5	70	5	miRanda, MirTarget, microT, PicTar, TargetScan
miR-125b-5p	80	5	35	2	miRanda, MirTarget, microT, PicTar, TargetScan
miR-133b	85	3	243	14	miRanda, MirTarget, microT, PicTar, TargetScan
miR-143-3p	51	2	39	24	miRanda, MirTarget, microT, PicTar, TargetScan
miR-145-5p	127	20	82	12	miRanda, MirTarget, microT, PicTar, TargetScan
miR-4454	0	0	0	0	miRanda, MirTarget, microT, PicTar, TargetScan
miR-4484	9	0	74	40	MirTarget, TargetScan

The biological function and potential functional pathways of each putative gene target were classified using the GO term and KEGG pathway. Since every gene is associated with many GO terms and KEGG pathways, the significant GO term (see Additional file 3) and KEGG pathway (see Additional file 4) for each miRNA were identified using Fisher’s exact test. Table 6 gives a few representative KEGG pathways for the putative target genes of the validated miRNAs as predicted by the miRFocus. The pathway analysis suggested that these miRNAs may potentially contribute to AF.

**Table 6:** A few representative KEGG pathways for the putative target genes of the validated miRNAs

**KEGG pathway**	**Target genes**	**miRNAs**
TGF-beta signaling pathway	ACVR1C, INHBB,SMAD1, SMAD3, TGFBR2,ZFYVE9	miR-26a-5p, miR-145-5p
MAPK signaling pathway	KRAS, MAP3K7, DUSP6, FLNB, PPP3CA, PRKX, RASA1, TGFBR2,MAP4K4, RAPGEF2	miR-143-3p, miR-145-5p
mTORsignaling pathway	EIF4E	miR-4484
VEGF signaling pathway	KRAS, SHC2	miR-143-3p, miR-4484
Calcium signaling pathway	ADCY3	miR-4484
Wntsignaling pathway	PLCB1, FRAT2, GSK3B, PPP3CB	miR-26a-5p
Gap junction	KRAS, ADCY3	miR-143-3p, miR-4484
Regulation of actin cytoskeleton	PFN2, ITGA6, ITGB8, KRAS	miR-133b, miR-143-3p
Hypertrophic cardiomyopathy (HCM)	IGF1, TPM3, TPM4	miR-1
Tight junction	MPP5, RRAS2, VAPA, PPP2CB, KRAS	miR-1, miR-23b-3p, miR-133b, miR-143-3p
Arrhythmogenic right ventricular cardiomyopathy (ARVC)	GJA1, ITGA6, SGCB, ITGB8, ACTB, ACTG1	miR-1, miR-30c-5p, miR-145-5p

## Discussion

Two recent studies investigating expression profiles of miRNA in mitral stenosis patients found the AF-associated miRNAs in RA [19] and LA [20], respectively, many of which were also found in our study. However, the studies investigated the alterations of miRNA expression profiles in relation to AF only in RA or LA tissue and they could not compare the potential differences of AF-associated miRNAs between the RA and LA. A recent study investigated changes in miRNA expression profiles in patients with valvular heart disease in relation to AF both in RA and LA tissue [28] and found the AF-associated miRNAs only in RA; the lack of detectable AF-associated miRNAs in LA may be partially due to lack of tissue availability; and the study also could not compare the potential differences of AF-associated miRNAs between the RA and LA. Thus, our study is the first to compare the potential differences of AF-associated miRNAs in the RA and LA from RMVD patients. We found that the development of AF in RMVD patients was associated with significant changes in miRNA expression in both RAA and LAA tissues, and these AF-associated miRNAs had different distributions in RAA and LAA. A total of 23 AF-associated miRNAs were both in RAA and LAA, while 45 AF-associated miRNAs were only in RAA, and 19 AF-associated miRNAs were only in LAA. The differential distributions of these AF-associated miRNAs may reflect different miRNAs mechanisms in AF between the RA and LA.

miRNA expression profiles are genetically programmed with temporal patterns (depending on developmental stage or disease status) [29]. Specific alterations in miRNA expression profiles are associated with specific disease pathophysiologies [30]. Our study has shown that AF status resulted in a larger number of miRNAs expressed as compared to the SR status (Figure 1B) and the AF-associated miRNAs were found in both RAA (Table 2, Figure 3A) and LAA (Table 3, Figure 3B) tissues from RMVD patients. Studies have shown that miRNAs may be involved directly or indirectly in AF by modulating atrial electrical remodeling (i.e. miR-1, miR-26, and miR-328) or structural remodeling (i.e. miR-30, miR-133, and mir-590). Yang et al. [31] reported that miR-1 overexpression slowed conduction and depolarized the cytoplasmic membrane by post-transcriptionally repressing KCNJ2 (potassium inwardly-rectifying channel, subfamily J, member 2; which encodes the K^+^ channel subunit Kir2.1) and GJA1 (gap junction protein, alpha 1, 43 kDa; which encodes connexin 43), and this likely accounts at least in part for its arrhythmogenic potential. Girmatsion et al. [32] indicated that miR-1 levels are greatly reduced in human AF, possibly contributing to up-regulation of Kir2.1 subunits, leading to increased cardiac inward-rectifier potassium current (I_K1_)_._ Luo et al. [13] identified miR-26 as a potentially important regulator of KCNJ2 gene expression and, via I_K1_, a determinant of AF susceptibility. Li et al. [12] reported that miR-133 and miR-30, as anti-fibrotic miRNAs [33,34], may play an important role in the control of structural changes in chronic AF. To date, the miR-23, miR-125, miR-143, miR-145, miR-4454, and miR-4484 have not been described as participating in AF pathology. Based on the predictions of putative target genes and pathways determined using miRFocus (Table 6), we have found that these miRNAs are potentially involved in several important biological processes and functional pathways associated with AF (e.g., TGF-beta, MAPK, VEGF, Calcium, Gap junction, mTOR, and Wnt signalling pathway, most of which have been implicated in the pathogenesis of AF). Thus, our results may implicate these miRNAs in the pathogenesis of AF.

miRNA expression profiles are also genetically programmed with certain spatial characteristics (depending on cell, tissue, or organ type) [35,36]. For example, miR-208 and miR-499 are cardiac-specific miRNAs exclusively expressed in cardiac tissues, while miR-1 and miR-133 are muscle-specific miRNAs preferentially expressed in cardiac and skeletal muscle [37,38]. We have found that the expression levels of most AF-associated miRNAs were identical between RAA and LAA tissues in SR patients, with exception of miR-23b-3p and miR-125b-5p (Figure 6). Thus, we conclude that most of the AF-associated miRNAs, except miR-23b-3p and miR-125b-5p, were not tissue dependent and the different distribution of these miRNAs in RA and LA is caused by the different mechanisms involved in AF between RA and LA. Our results have shown that miR-23b-3p was more highly expressed in the SR-LAA group than in SR-RAA and was AF-associated miRNAs only in LAA tissue, while, miR-125b-5p was more highly expressed in the SR-RAA group than in SR-LAA and was AF-associated miRNAs only in RAA tissue. miR-23b-3p and miR-125b-5p are tissue dependent and they may play a role in the progress from SR to AF as decided by the initial expression (SR status) in RAA and LAA. The different expression of miR-23b-3p and miR-125b-5p between RAA and LAA in RMVD patients with SR maybe due to RMVD, because Cooley et al. [28] reported that some miRNAs expression changes in RA and LA with the development of valvular heart disease. In addition, in RMVD patients, the association between LA size and AF is well established and LA dilatation is considered both a cause and consequence of AF [15]. Our results have shown that LA size of the patients with AF was significantly greater than patients with SR (Table 1), thus, it is possible that the significant structural remodelling of the LA also alters the microRNA expression profiles and cause, at least in part, the different distributions of AF-associated miRNAs in the RA and LA from RMVD patients.

The main limitation of this study was the small number of patients included. This was due, in part, to the difficulty of finding RMVD patients with SR. Second, We could not conduct experiments to modulate miRNA levels in native human tissues. Hence, the evidence presented here is not a direct cause and effect relationship. Furthermore, the exact targets and pathways of AF-associated miRNAs causing AF in RMVD patients remain elusive and deserve further investigation [19]. Finally, the patients included in this study were a specific cohort with preserved systolic left ventricular function and little comorbidity; they were undergoing mitral valve replacement surgery. Changes identified in this population may not be representative of other cohort of populations [32].

## Conclusions

We have found the different distributions of AF-associated miRNAs in the RAA and LAA from RMVD patients. This may reflect different miRNA mechanisms involved in AF between the RA and LA. These findings may provide new insights into the underlying mechanisms of AF in RMVD patients and provide potential novel mechanism-based therapeutic strategies for AF.

## Abbreviations

AF: Atrial fibrillation; SR: Sinus rhythm; RMVD: Rheumatic mitral valve disease; MiRNA: microRNA; RA: Right atrium; LA: Left atrium; RAA: Right atrial appendage; LAA: Left atrial appendage; ECG: Electrocardiogram; NYHA: New York Heart Association; LVEF: Left ventricular ejection fraction; PGR: Photogene.rated reagent; CV: Coefficient of variation; qRT-PCR: Quantitative real-time PCR; GO: Gene Ontology; KEGG: Kyoto Encyclopedia of Genes and Genomes.

## Competing interests

The authors declare that they have no competing interests.

## Authors’ contributions

HL carried out the molecular studies, participated in the sequence alignment and drafted the manuscript. HQ, GXC, MYL, JR, JPY participated in open heart surgery, and collected clinical samples. HL, ZKW participated in the design of the study and performed the statistical analysis. ZKW, HQ, GXC conceived the design of the study, and participated in its implementation and coordination, and helped to draft the manuscript. HL, HQ, and GXC contribute equally to this article. All authors have read and approved the final manuscript.

## Supplementary Material

Additional file 1 Primers for qRT-PCR of miRNA and RNU6B. Click here for file

Additional file 2 Prediction of putative target genes of the validated miRNAs. Click here for file

Additional file 3 Biological processes of the predicted miRNA targets. Click here for file

Additional file 4 Pathway analysis of the validated miRNAs. Click here for file
